# The diagnosis and treatment strategy of occipital skull mass in hemophilic patients: a rare case report and literature review

**DOI:** 10.1186/s41016-019-0155-x

**Published:** 2019-04-03

**Authors:** Qingyuan Liu, Jun Wu, Chunde Li, Shuo Wang

**Affiliations:** 1 Department of Neurosurgery, Beijing Tiantan Hospital, Capital Medical University, No.119 South 4th Ring West Road, Fengtai District, Beijing, 100070 China; 2 China National Clinical Research Center for Neurological Diseases, Beijing, People’s Republic of China; 3 Center of Stroke, Beijing Institute for Brain Disorders, Beijing, People’s Republic of China

**Keywords:** Hemophilic pseudotumor, Perioperative management, Diagnosis strategy, Replacement treatment, One-stage cranioplasty

## Abstract

**Background:**

Cranial hemophilic pseudotumor (cHPT) is a very rare disease, which is easy to misdiagnose. It is also difficult to manage such patients. We reported the first case of occipital cHPT.

**Case presentation:**

Here, we presented a rare case of an occipital bone mass in a 3-year-old boy who was diagnosed with hemophilia A. The mass was misdiagnosed as an aneurysmal bone cyst by pathological examination. After resection, the patient underwent one-stage cranioplasty. However, the patient was admitted again for hematoma caused by an invasive procedure. A second surgery and one-stage cranioplasty were performed at the same time. A follow-up 3 months after discharging showed the patient was uneventful, and the titanium mesh was well fixed.

**Conclusion:**

The diagnosis of cHPT requires the combining of history, radiological examination, and pathological examination. Resection is the best choice for symptomatic cHPT. Replacement treatment and less invasive treatment can make perioperative management safer. One-stage cranioplasty for resection of an occipital cHPT can improve the quality of life.

## Background

Hemophilia could cause severe hemorrhage complication but is rare in the skull. The cranial hemophilic pseudotumor (cHPT) is a severe complication, which occurs in 1–2% of hematoma in hemophilic patients [1, 2]. It is easy to misdiagnose and delay the treatment because of several similar diseases, such as the aneurysmal bone cyst (ABC). [3–5]. Perioperative management and neurosurgical intervention for hemophilic patients are both challenges [2, 4, 6]. In the following cases, we presented a case of HPT patient and describe our diagnosis and treatment experience in this disease.

## Case presentation

A 3-year-old boy was admitted for progressively enlarging occipital mass and persistent headache. The mass was found 1 year ago and grew obviously within 2 months. The child had a traumatic history in the occipital (about 1.5 years ago).

**Fig. 1:**
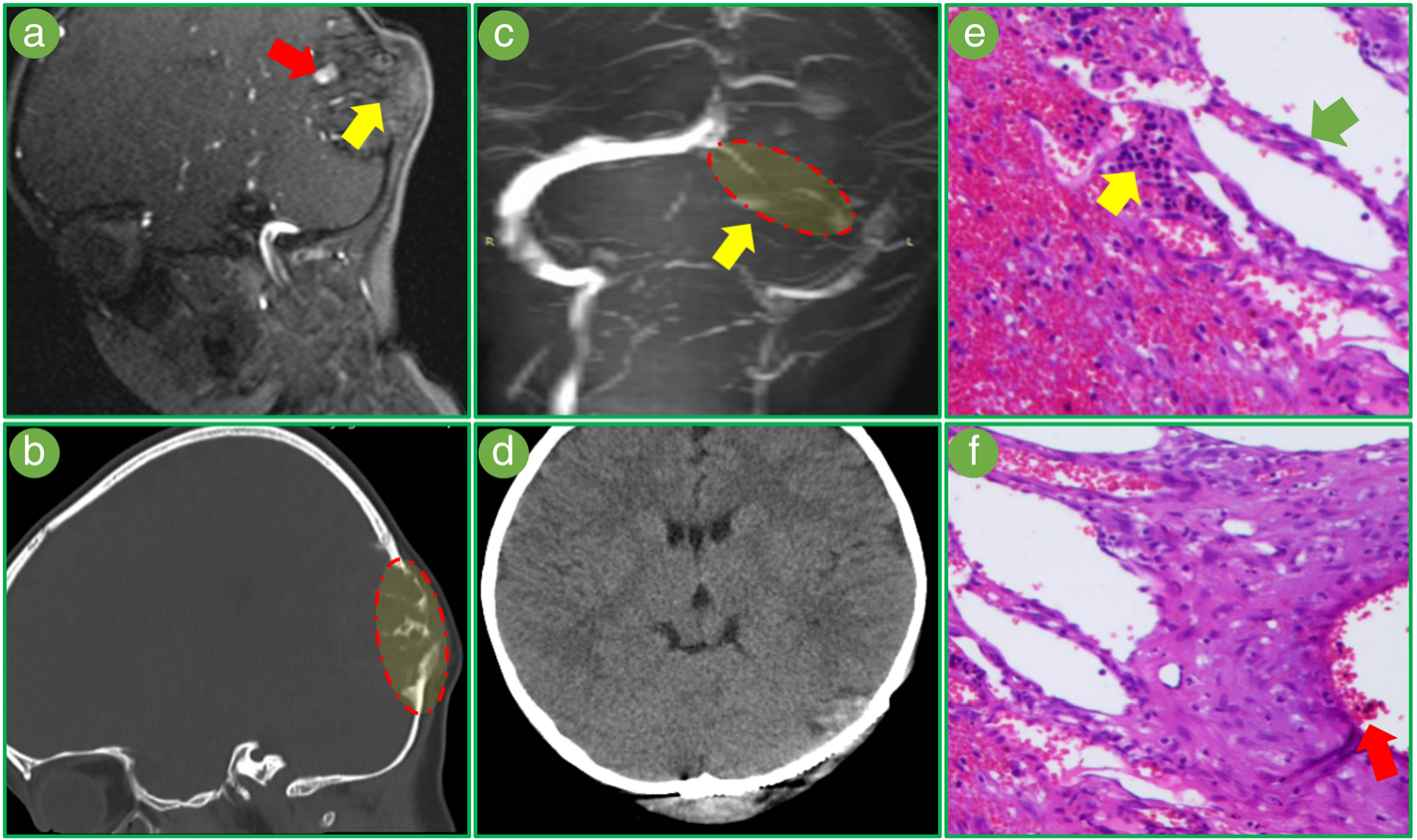
**a** MRA showed a poor arterial supply (yellow arrow). The artery closest to the lesion is located outside the lesion (red arrow). **b** CT suggested an irregular destruction of the occipital bone and small cysts within the lesion. **c** MRV showed the left transverse sinus was blocked totally (yellow arrow). **d** The postoperative CT was negative, no obvious sign of bleeding. **e** Pathological examination found “giant cells” around the cysts (green arrow), which should be hemosiderin-laden macrophages. We can see that these cells are scattered within the lesions (yellow arrow). **f** There were numerous cystic spaces (blue arrow) containing bloody fluid (red arrow) in the lesion, which is easily mistaken as an ABC

Magnetic resonance angiography (MRA) did not find obvious arterial supply within the lesion (Fig. 1a). CT suggested an irregular destruction of the occipital bone and small cysts within the lesion (Fig. 1b). Magnetic resonance venography (MRV) showed that the left occipital sinus was blocked totally (Fig. 1c).

Laboratory examination on admission suggested that the activated partial thromboplastin time (APTT) was prolonged (64 s), and the clotting factor VIII was decreased (48.4%). The hematologist considered the patient as a hemophiliac. A replacement treatment was subsequently performed using massive fresh frozen plasma (FFP). After controlling the APTT within 11.6 to 15.9 s, the coagulation was subsequently monitored for 2 days. Confirming that the coagulation was suitable for surgery, surgical resection was immediately arranged.

What we saw intraoperatively was an organizing hematoma with scattered calcification, massive fibrous tissue hyperplasia around the lesion, and several small bony cysts within the lesion. After total resection, one-stage cranioplasty was performed too. The volume of intraoperative bleeding was 300 ml. Postoperative CT was negative (Fig. 1d). After the operation, we continuously monitored the coagulation condition. On the third day, a pinpoint errhysis and prolonged APTT (32 s) were found. A replacement treatment was performed immediately using FFP. No signs of hemorrhage were subsequently seen until discharge.

The final pathological diagnosis was cranial hemophilic pseudotumor (Fig. 1e, f).

**Fig. 2:**
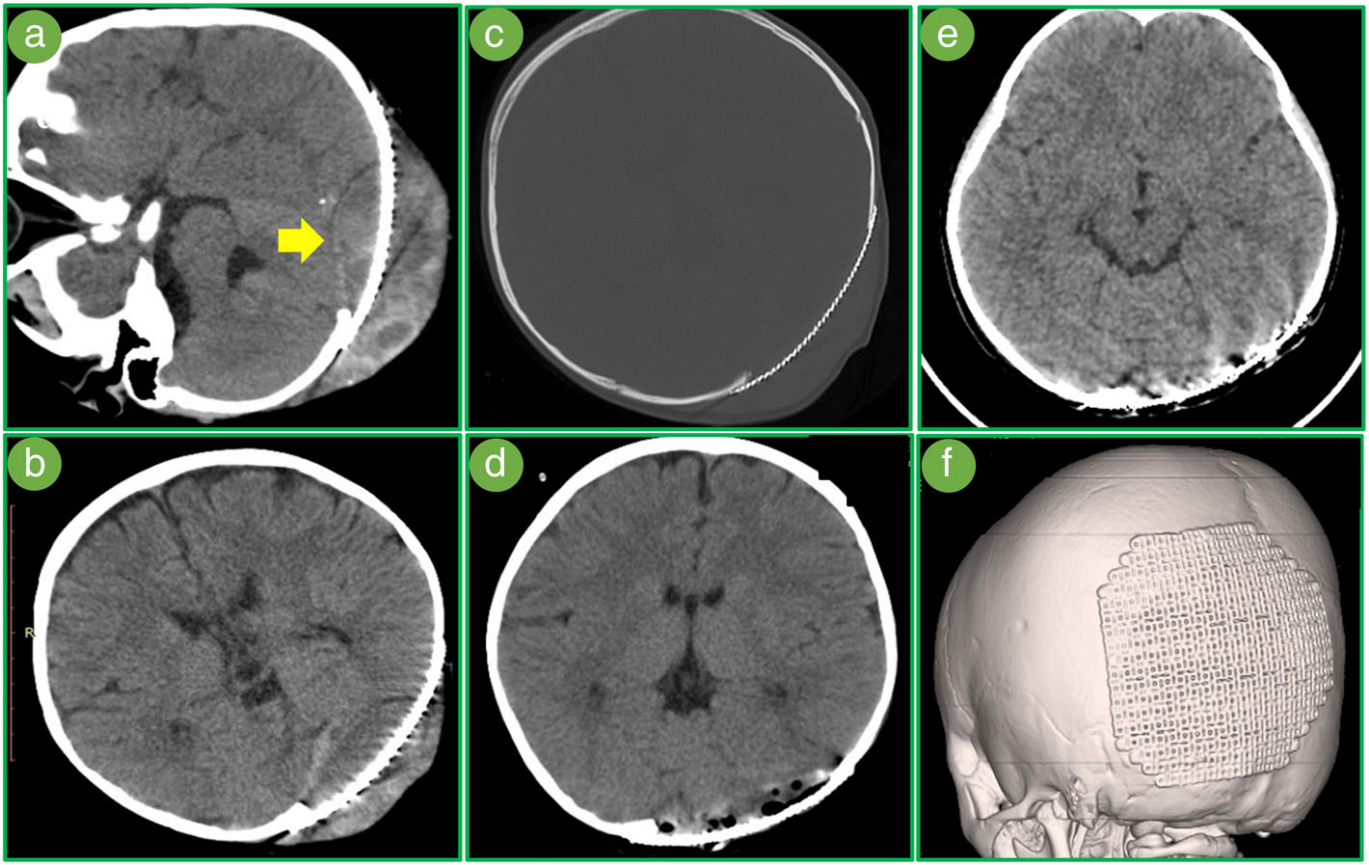
**a** After puncturing, a compression caused by epidural hematoma was found on CT (yellow arrow). **b** Postoperative CT found a hematoma (24 h after surgery). **c** The mesh was fixed well. **d** Four days after surgery, hematoma was obviously absorbed. **e**, **f** Three months of surgery, the mesh was well fixed, no bleeding and subcutaneous hydrops

The patient was admitted for subcutaneous hydrops 12 days after discharge. A puncture was performed. One hour later, the patient felt nausea and headache. A compression and sign of cerebral hernia caused by epidural hematoma was found on CT (Fig. 2a). A coagulation examination showed coagulation function was catastrophic (APTT 50.3 s, factor VIII 32.1%). Thus, a replacement treatment was performed until the APTT was significantly improved (21.6 s). A surgery was immediately performed for cerebral hernia. Intraoperatively, plasma transfusion was continued. A one-stage cranioplasty was subsequently performed after evacuating the hematoma. The magnitude of intraoperative bleeding was 500 ml. Postoperatively, the APTT was still abnormal but no signs of bleeding were found. A hematoma was still visible on CT but showed no signs of enlargement (Fig. 2b). The mesh was fixed well (Fig. 2c). On the third day after the operation, we found that APTT (39.6 s) was prolonged again. A replacement treatment was then performed using FFP. Four days after surgery, hematoma was significant absorbed (Fig. 2d) and the APTT was improved (32.7 s). The wound was healing well.

Three months after discharging, a radiological follow-up showed the titanium mesh healed well with surrounding tissue without subcutaneous hydrops (Fig. 2e, f). The patient can live as normal as before the operation without much change in appearance and neurological deficit.

## Discussion

The cHPT is so rare that only seven cases of this disease have been reported (Table 1). Here, we reported the first case of occipital cHPT.

**Table 1:** The result of literature review about cases of hemophilic pseudotumor

First author	Year	Deficit factor	Location	Size	Traumatic history
Killby D [20]	1972	VIII	Temporal	5–6 cm	Y
Horton DD [12]	1993	VIII	Parietal	Non	Y
Sim KB [13]	1996	VIII	Parietal	5 × 2 cm	Y
Conde F [6]	2006	VIII	Parietal	5 × 3 cm	N
Inoue T [2]	2008	IX	Frontal	> 3 cm	N
Zafar T [9]	2008	VIII	Frontal	20 × 16 cm	Y
Kashiwazaki [10]	2012	VIII	Temporal	Non	N
Zhang [21]	2014	VIII	Bi-temporal	Non	Y
Our present case	2017	VIII	Occipital	9 cm	Y

**Fig. 3:**
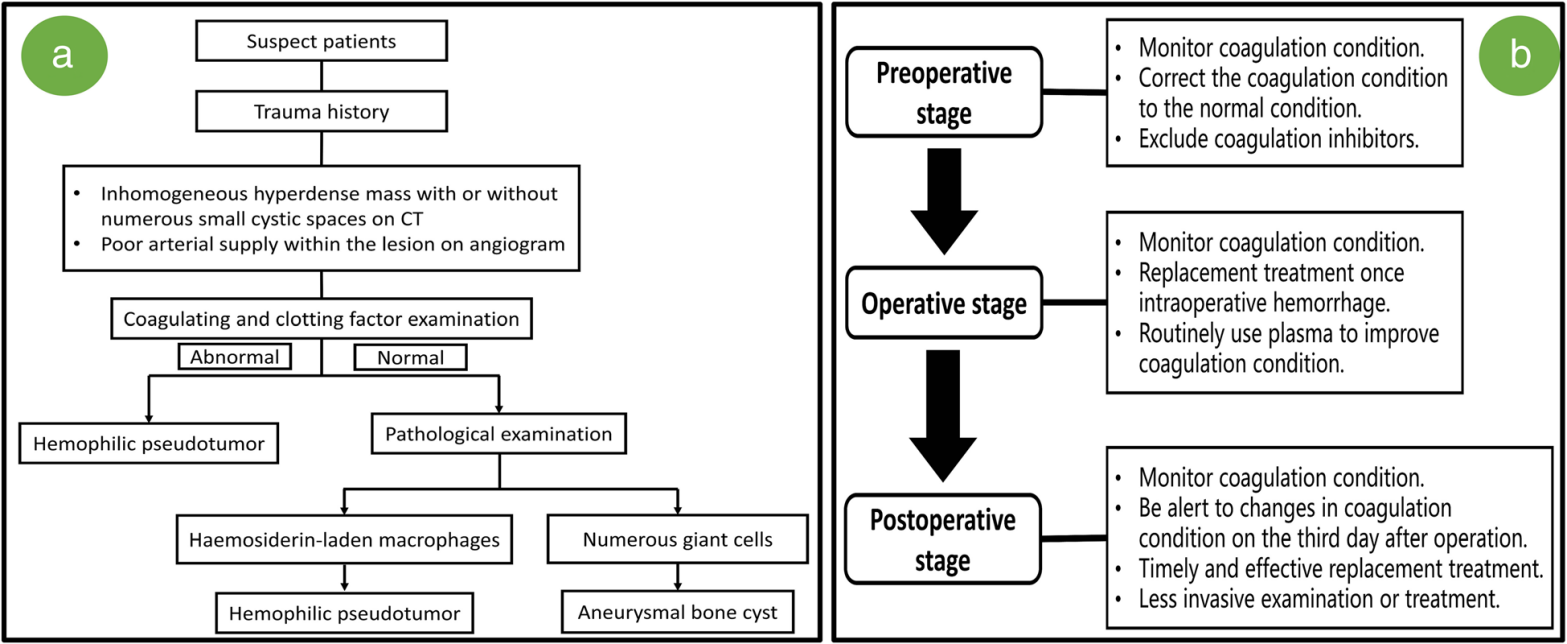
**a** Our diagnosis experience was summarized here. Once encountering suspicious patients, the patient should first be identified with a trauma history and complete the radiological examination (mainly CT and angiogram). Coagulation examination is not only helpful for the diagnosis of hemophilia, but also for the diagnosis of hemophilic pseudotumor. The key point of pathological examination is to distinguish hemosiderin-laden macrophages from giant cells. **b** Our perioperative management experience was summarized here. Monitoring of the coagulation condition should be carried out throughout the perioperative management. The key point for preoperative stage is coagulation correction, and the key point for postoperative stage is timely replacement treatment and less invasive examination/treatment

It is difficult to diagnose a cHPT for similar features between cHPT and some diseases, such as ABC [7, 8]. They could both present as a progressively enlarging mass and the neurological deficit caused by the compression. The diagnosis of cHPT may be performed by radiological examination, which is defined as bone destruction and bony cyst containing fluid [9, 10], whereas ABC could also present as similar features [7]. Even on histology, cHPT and ABC can share similar features, such as single fibrous capsule or multiple bony cysts containing bloody fluid [7, 9, 11-13]. However, we can still identify a cHPT by some features. Previous cases showed 5/8 of patients with cHPT had a history of head trauma, and a hematoma often occurred after injury but were often not well treated [2, 4, 10, 12, 13]. One year later, there will be a progressively enlarging mass within the injury region [2, 9]. Meanwhile, attention should be paid to history of hemophilia and relevant examination, which is not only important for treatment, but also important for diagnosis [9]. A CT reveals numerous small cysts, and an angiogram shows the presence of poor arterial supply [7, 9], which could exclude the ABC. As for histology, it is very important for cHPT to recognize the hemosiderin-laden macrophages, which is easily mistaken as atypical giant cells [9]. This is the main reason for the misdiagnosis of our case. The cyst walls were lined by giant cells for ABC [11], whereas hemosiderin-laden macrophages were dispersed within the lesions. Moreover, the ABC usually contains large cysts with obvious arterial structure [11], whereas the cHPT usually contains many small cysts with significantly organized hematoma and massive inflammatory cells infiltration [9]. However, it is difficult to diagnose the cHPT based on single clinical features, and the above characteristics should be comprehensively considered. Fig. 3a summarized our experience of diagnosis for the cHPT.

The surgical resection is the preferred treatment [14–16]. However, this treatment is only considered when patients have symptoms in order to decrease the mortality and morbidity [6, 16]. Whether the patient needs surgical intervention or not, replacement is an important treatment. Correction of coagulation to the normal can reduce the risk of intraoperative hemorrhage. This is especially important for child patients because hemorrhage can be fatal. Significant decrease of coagulation factors may occur on the third day after surgery, which should be monitored in time. FFP may need much more than the recombinant factor to reach the same effect [17, 18]. However, considering the recombinant factor is expensive and difficult to acquire, FFP is the first-line option. Avoid invasive operation in the case of unknown coagulation function. In our case, a puncture was performed in absence of coagulation function, which subsequently resulted in a hematoma. However, examination of coagulation function should be executed firstly if an invasive procedure is inevitable. Fig. 3b summarized our experience of management strategy for the cHPT.

Due to the risk of postoperative bleeding and little impact on the quality of life, no one-stage repair has been reported in previous cases [2, 4, 10, 12, 13]. However, the resection of occipital cHPT will leave a bone deficit, which could hugely impair the patients’ living quality because of special sleeping position and easily hurting by external force. Thus, we recommended one-stage cranioplasty, especially for occipital cHPT. In addition to improving the quality of life, a one-stage cranioplasty could improve psychological status and neurocognitive function [19]. We believed that these advantages were also confirmed by our present case. It can be seen that under good control of coagulation condition, one-stage cranioplasty is a very good choice for occipital lesions.

## Conclusion

The diagnosis of cHPT requires the combining of history, radiological examination, and pathological examination. Resection is the best choice for symptomatic cHPT. Replacement treatment and less invasive treatment can make perioperative management safer. One-stage cranioplasty for resection of occipital lesion can improve the quality of life.

## Abbreviations

ABC: Aneurysmal bone cyst
APTT: Activated partial thromboplastin time
cHPT: cranial hemophilic pseudotumor
CT: Computed tomography
FFP: Fresh frozen plasma
MRA: Magnetic resonance angiography
MRV: Magnetic resonance venography
